# Olecranon Fossa Fenestration Approach to the Coronoid and Anterior Elbow: The Way OFF.

**DOI:** 10.1016/j.eats.2024.103078

**Published:** 2024-06-18

**Authors:** Paolo Arrigoni, Valeria Vismara, Alfonso Liccardi, Jane Messina, Pietro Simone Randelli

**Affiliations:** a I Clinica Ortopedica, Azienda Socio Sanitaria Territoriale Centro Specialistico Ortopedico Traumatologico Gaetano Pini-CTO, Milan, Italy; b Scuola Di Specializzazione in Ortopedia e Traumatologia Università Degli Studi Di Milano, Milan, Italy; c Laboratory of Applied Biomechanics, Department of Biomedical Sciences for Health, Università Degli Studi Di Milano, Milan, Italy; d U.O.C. 1°Clinica Ortopedica, ASST Centro Specialistico Ortopedico Traumatologico Gaetano Pini-CTO, Milan, Italy; e Research Center for Adult and Pediatric Rheumatic Diseases (RECAP-RD), Department of Biomedical Sciences for Health, Università Degli Studi Di Milano, Milan, Italy

## Abstract

Hypertrophic osteoarthritis of the elbow is a challenging condition that can vary from mild to severe, affecting patients’ quality of life due to pain and loss of range of motion. A consensus about its treatment does not exist. Open arthrolysis with capsular release, synovectomy, Outerbridge-Kashiwagi fenestration, and removal of loose bodies and osteophytes demonstrated good results. In more recent times, an arthroscopic procedure has been shown to have the same efficacy as the open one. The aim of this Technical Note is to describe an all-arthroscopic procedure to reach the coronoid tip, in a safe and reproducible manner, through an olecranon fossa fenestration with a direct transtricipital posterior portal and an anterior cruciate ligament guide.

Hypertrophic osteoarthritis of the elbow is a challenging condition that can vary from mild to severe, affecting patients’ quality of life. It is characterized by the abnormal growth of bone tissue, leading to joint inflammation and degeneration. Various conditions play a role in its development, including age-related wear and tear; repetitive stress on the joint, such as in athletes or people involved in high physically demanding occupations; and multiple pathologic conditions like congenital malformations, hemophiliac arthritis, and infection. Patients complain of pain, crepitus, paresthesia, and synovitis and often of elbow stiffness.^1^^,^^2^ Elbow’s functional range of motion (ROM) is 30° to 130° of flexion-extension and –50° to 50° of prono-supination. Loss of ROM can be due to the bones or soft tissues. Intrinsic and extrinsic causes of elbow stiffness have been described and often coexist.^3^, ^4^, ^5^ Intrinsic causes include heterotopic ossification, loose bodies, intra-articular adhesions, malalignment, and osteochondral defect and refer to intra-articular pathologic conditions, whereas subcutaneous tissue, capsule, collateral ligament, and skin contractures account for the extrinsic causes.^6^^,^^7^

Although there have been many studies of hypertrophic osteoarthritis of the elbow, there is still no consensus about its treatment. Conservative management includes anti-inflammatory drugs, physical therapy, static or dynamic splinting, casting, manipulation (this procedure has been abandoned by most surgeons because of its risk of complication and development of heterotopic calcifications), and injection of botulin toxin A. Recalcitrant cases, inability to achieve functional ROM, and poor quality of life have been considered indications for surgery. Historically, open arthrolysis with capsular release, synovectomy, Outerbridge-Kashiwagi fenestration, and removal of loose bodies and osteophytes demonstrated good results.^8^, ^9^, ^10^ In 1993, Jones and Savoie^11^ were the first to describe an all-arthroscopic procedure that, over the years, has been shown to have the same efficacy as the open one. The aim of this Technical Note is to describe an all-arthroscopic procedure to reach the coronoid tip, in a safe a reproducible manner, using a trans-coronoid fossa portal and an anterior cruciate ligament (ACL) guide.

## Surgical Technique

This procedure is carried out in accordance with the Code of Ethics as described by the Declaration of Helsinki.

### Position and Setup

The patient is set in a modified lateral decubitus position, with the operative arm at 100° flexion of the shoulder with the use of an arm holder. The elbow is positioned in 90° of flexion, with the forearm hanging free to gravity. The elbow joint is distended with 20 to 30 cc of saline solution. The posterior compartment is assessed first, with the elbow at 70° of flexion.

### Step 1: Diagnostic Elbow Arthroscopy

**Fig 1:**
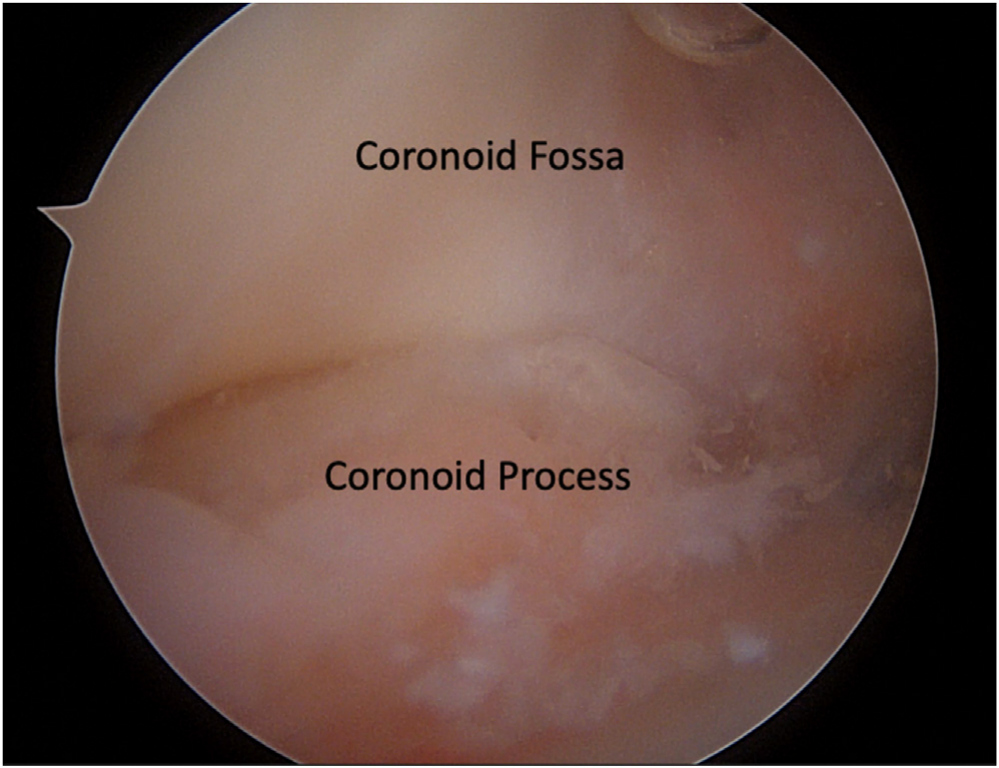
Intraoperative image of the right elbow: anterior chamber diagnostic evaluation, performed with the scope in the AM portal. The coronoid fossa and coronoid process can be identified. Due to the underlying pathology, hypertrophic osteoarthritis, both structures have lost their natural margins and appear deformed. (AM, anteromedial.)

After a first phase into the posterior room looking from the posterolateral portal, a 5.5-mm shaver is inserted through the transtricipital posterior (TTP) portal. After identification of the native bone and stripping off the posterior capsule, the scope is shifted in the TTP portal, and the shaver is inserted in the posterolateral one, to expand the olecranon recess. The anterior room is then evaluated though the anteromedial (AM) and anterolateral (AL) portals, which allow identification of the joint surface (Fig 1).

### Step 2: Anterior Osteophyte Removal

**Fig 2:**
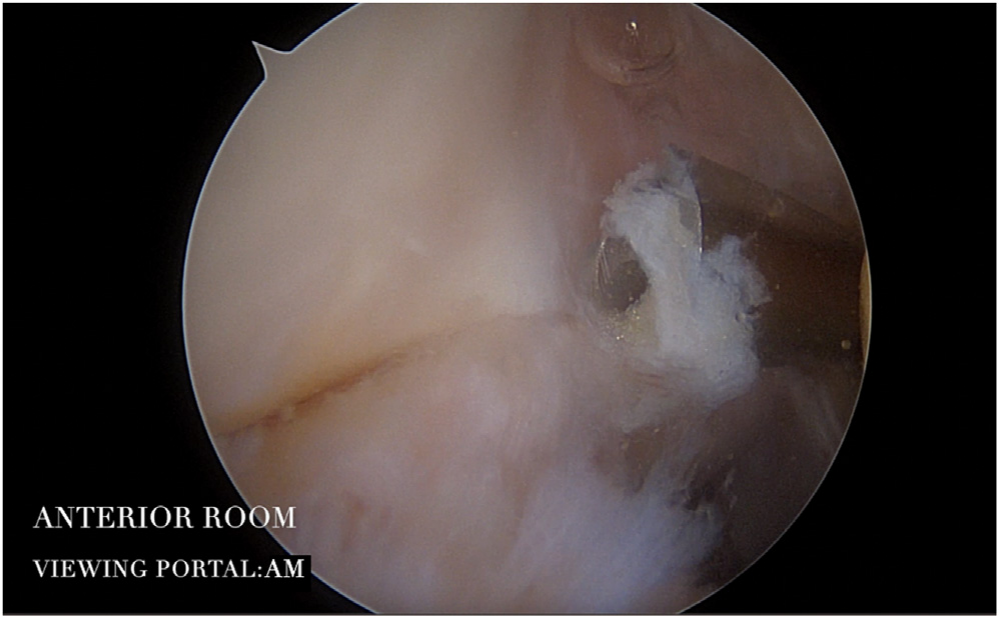
Intraoperative image of the right elbow: reshaping of the coronoid fossa with a helicoidal-shaped burr (Stryker Instruments), inserted through the AL portal. The scope is inserted in the AM portal. (AL, anterolateral; AM, anteromedial.)

By holding the shaver in the AM portal, osteophytes can be removed from the radial fossa, whereas by holding the shaver in the AL portal, osteophytes can be removed from the coronoid fossa. This allows re-creating the normal anatomy of the coronoid fossa, which is often filled with new bone (Fig 2).

### Step 3: ACL Guide Insertion

**Fig 3:**
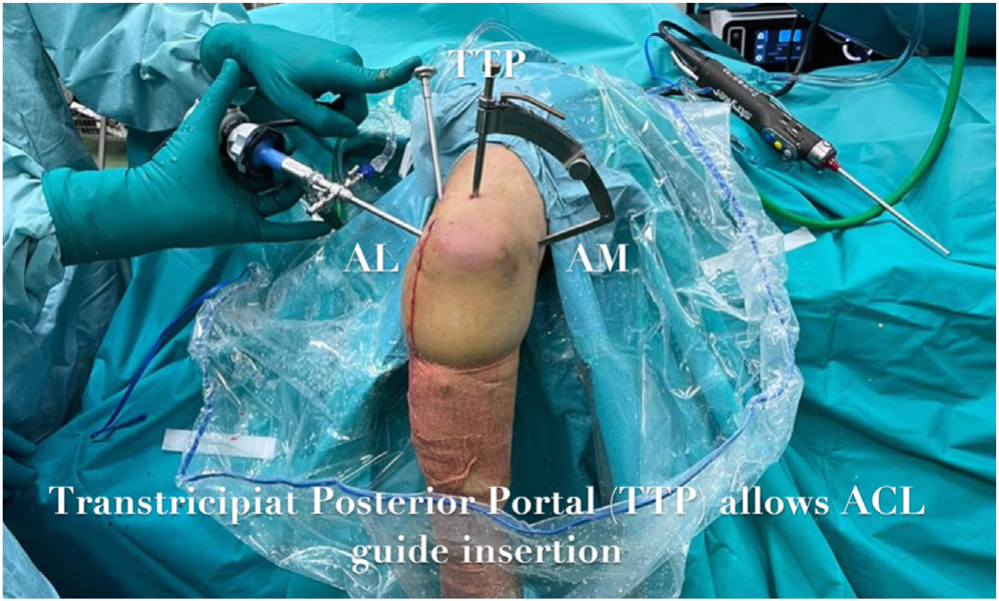
ACL guide setup of the right elbow: the aimer of the guide enters from the AM portal while the cannulated cannula passes through the TTP portal. The scope is inserted in the AL portal. A retractor is used in the proximal AL portal to allow a better arthroscopic visualization. (ACL, anterior cruciate ligament; AL, anterolateral; AM, anteromedial; TTP, transtricipital posterior portal.)

**Fig 4:**
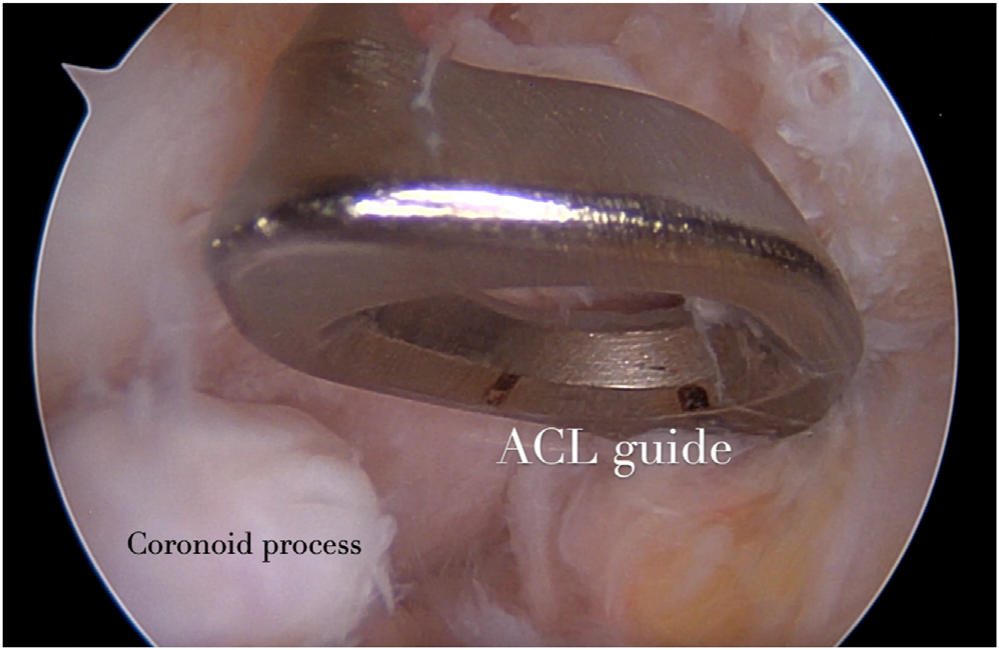
Intraoperative view of the ACL guide of the right elbow: the ACL guide aimer enters through the AM portal, and it is positioned in the center of the coronoid fossa. (ACL, anterior cruciate ligament; AM, anteromedial.)

With the scope in the AL portal, a femoral ACL guide (Arthrex) is used to develop the olecranon fenestration, with the elbow at 110° of flexion. The aimer of the guide enters from the AM portal while the cannulated cannula for the 2.0 Kirshner wire (K-wire) passes through the TTP portal. Often a retractor is used in the proximal AL portal to allow better arthroscopic visualization and range of movement (Figs 3 and 4).

### Step 4: Olecranon Fossa Fenestration Development

**Fig 5:**
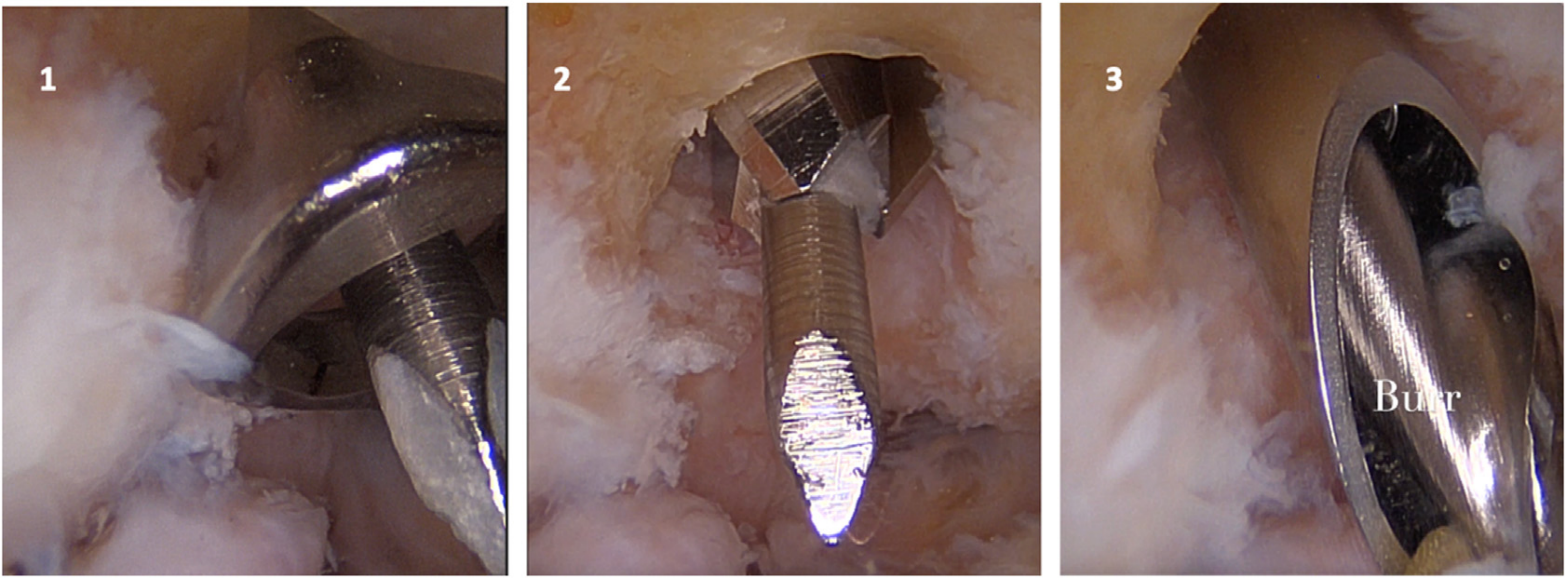
Intraoperative sequence of olecranon fossa fenestration development: (1) a K-wire is then drilled through the ACL guide; (2) humeral fenestration is developed through the ACL drill, guided by the K-wire; and (3) a helicoidal-shaped burr goes through the olecranon fenestration to complete coronoid fossa enlargement. Throughout the procedure, the scope is maintained in the AL portal. (ACL, anterior cruciate ligament; AL, anterolateral.)

A K-wire is then drilled through the ACL guide, in the center of the coronoid fossa, and a humeral fenestration is developed with an ACL cannulated drill (6.0) first, which goes over the K-wire. To prevent the K-wire from damaging the surrounding structures, when pushed down by the ACL cannulated drill, often a retractor is inserted from the AM portal. Once the fenestration is complete, a bony helicoidal-shaped burr (Stryker Instruments) can enter the hole through the olecranon fossa and reach the conoid fossa. The fenestration is enlarged though the use of the burr (Fig 5).

### Step 5: Working Through the TTP Portal

**Fig 6:**
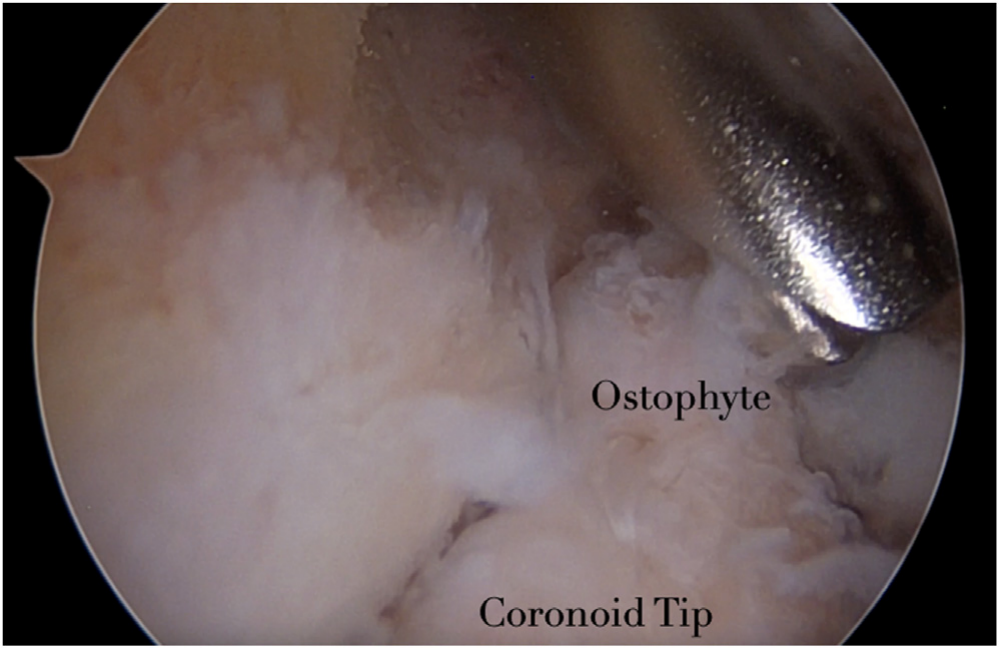
Intraoperative view of the right elbow: with the scope in the AL portal and the burr in the TTP, flexion of the elbow brings the coronoid tip in closer proximity to the humeral fenestration. Osteophytes of the tip of the coronoid can be removed. (AL, anterolateral; TTP, transtricipital posterior.)

Through this olecranon fossa fenestration working from the direct TTP portal, coronoid osteophytes can be very well addressed in a safe and reproducible manner. Coronoidoplasty and coronoid fossa enlargement is then performed with a helicoidal-shaped burr. Flexion of the forearm brings the coronoid tip in closer proximity to the olecranon fossa fenestration. This allows decreasing the distance between the burr and the coronoid tip, achieving a greater osteophyte exposure and performing a more accurate procedure (Fig 6 and Video 1).

## Discussion

Arthrolysis is often performed in the setting of elbow hypertrophic osteoarthritis and stiffness, with the goal of achieving better ROM and relieving pain. Elbow arthroscopy has always been considered more challenging compared with other anatomic districts due to the proximity of the joint with important anatomic structures, mainly nerves and vessels. Complications rates are considerably higher compared with other joints and add up to 10% to 20%.^8^^,^^12^ In the past 2 decades, its indications have expanded. One of the main difficulties of performing this surgery, especially at the beginning of the learning curve, is timing. Often a pneumatic tourniquet is applied before the procedure starts, so timing is of essence. A systematic approach allows surgeon to decrease the complication rates and decrease surgery time. The Outerbridge-Kashiwagi procedure has been described to treat mild to moderate osteoarthritis of the elbow. A tunnel is created through the olecranon fossa to reach the tip of the coronoid with a posterior approach and to avoid impingement between the tip of the coronoid and the olecranon with the olecranon fossa during ROM. Arthroscopic fenestration of the olecranon fossa to achieve the same results has already been proposed by several authors. The drilling is performed by freehand, and a great portion of bone is removed to access the anterior elbow compartment.^13^^,^^14^ We described an all-arthroscopic way to reach the coronoid process, which is safe and reproducible. Often osteophytes located at the level of the coronoid process are difficult to address, coming from either the AM or the AL portal. Addressing the coronoid process from upward is more intuitive. On the other hand, developing a humeral tunnel without the help of a guide is often difficult if not in the hands of expert surgeons. This technique allows the surgeon to reach the tip of the coronoid in safe and reproducible manner; enlarging the coronoid fossa allows delayed contact of the coronoid tip, theoretically increasing ROM; and the use of an ACL guide proved to be effective in the development of a humeral tunnel located just above the coronoid process. For this reason, we think that this tool could be helpful in the hands of less experienced surgeons and could save time during a complex arthroscopic procedure. Indeed, this technique adds steps to the overall procedure but may avoid the need for open arthrolysis in more complex cases, and an ACL guide is needed to aim at the center of the coronoid process without damaging the surrounding structure, especially in less experienced hands. Further developments of this technique may involve coronoid fracture fixation and loose body removal (Tables 1 and 2).

**Table 1:** Advantages and Disadvantage of the WAY OFF Technique

The WAY OFF Advantages	The WAY OFF Disadvantages
The tip of the coronoid process can be reached in a safe, fast, and reproducible fashion.	The procedure adds further steps to arthroscopic surgery.
The enlarging of the coronoid fossa allows delayed contact of the coronoid tip, theoretically increasing ROM.	An ACL guide is needed, especially in less experienced hands, to aim at the center of the coronoid process without damaging surrounding structures.
The use of a femoral ACL guide proved to be effective in the development of a humeral tunnel located just above the coronoid process.	

ACL, anterior cruciate ligament; ROM, range of motion.

**Table 2:** Pearls and Pitfalls of the WAY OFF Technique

The WAY OFF Pearls	The WAY OFF Pitfalls
Enhanced precision in reaching the tip of the coronoid process, crucial to facilitate coronoid-plasty	Using an ACL guide is time-consuming, and the procedure’s duration is frequently constrained by the tourniquet time limit
Improved arthroscopic triangulation is achieved more easily in a 90° configuration (the scope in the AL portal and the burr in the TPP portal), rather than in the 180° configuration (the scope in the AL portal and the burr in the AM portal)	Availability of ACL guide and expertise in its use
Decreased risk of additional osteochondral damage with the burr	Drilling a hole at the level of the olecranon fossa, which could otherwise be left untouched
Concomitant coronoid fossa enlargement	

ACL, anterior cruciate ligament; AL, anterolateral; AM, anteromedial; TTP, transtricipital posterior.

The use of a trans-coronoid fossa approach and an ACL femoral guide to create an olecranon fossa fenestration allows the surgeon to reach the tip of the coronoid process in a safe and reproducible fashion. At the same time, enlarging the coronoid fossa allows delayed contact for the coronoid tip.

## Supplementary Data

Video 1 A way to approach the anterior room of the elbow (right elbow), coronoid process. The TTP portal allows ACL guide insertion (6.0 ACL drill and burr to enlarge the coronoid fossa), with easier coronoid-plasty by elbow flexion. (ACL, anterior cruciate ligament; AL, anterolateral; AM, anteromedial; TTP, transtricipital posterior.)
